# Supplementary material for: Dissecting esophageal squamous-cell carcinoma ecosystem by single-cell transcriptomic analysis
Source: Nat Commun. 2021 Sep 6;12:5291. doi: 10.1038/s41467-021-25539-x (PMC8421382; doi:10.1038/s41467-021-25539-x)
Supplement: Supplementary file 1 — Supplementary Information [file 41467_2021_25539_MOESM1_ESM.pdf]

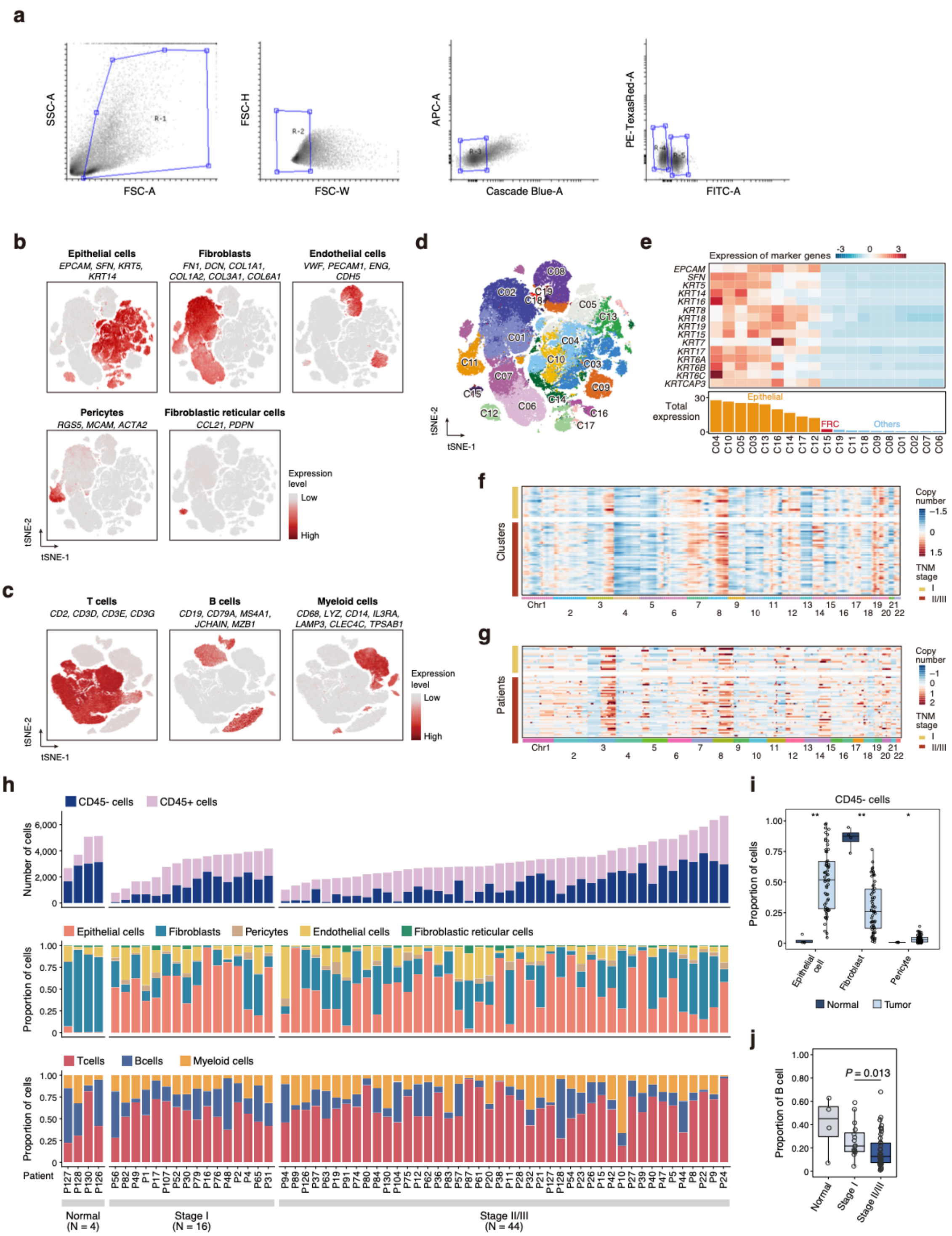

Supplementary Fig. 1 Identification of cell types and sample statistics. a Scaterplots showing gating strategy of flow cytometry and cell sorting. R-1, R-2 and R-3 were to exclude cellular debris, doublets and dead cells, respectively. R-4 and R-5 were to sort cells by FITC-labeled CD45 antibody. **b** and **c** The integrated expression levels of the marker genes for each major cell type in all CD45- and CD45+ cells. **d** tSNE plot of all CD45- cells, colored by clusters. **e** Heatmap showing the expression levels of 15 typical epithelial marker genes in all CD45- cell clusters (*top*) and the histogram showing aggregated expression level of all markers (*bottom*). **f** Heatmap showing CNVs inferred from single cell transcriptome dataset. The samples are ordered by stage. Red: amplification; blue: deletion. **g** Heatmap showing CNVs calculated from whole genome sequencing analysis. **h** Stacked histograms showing the cell numbers and proportions of major cell types

identified in both CD45- and CD45+ cells. **i** Boxplots displaying the different proportions of cell types between ESCC tumor (N = 60) and adjacent normal (N = 4) tissues (two-sided Wilcoxon tests; *: $P < 0.05$, **: $P < 0.01$). *P* values: 0.001 (epithelial cell), 0.001 (fibroblast), 0.010 (pericyte). **j** Boxplots comparing proportion of B cells in normal tissues (N = 4), stage I (N = 16) and stage II/III (N = 44) tumors (two-sided Wilcoxon tests). Box plots in (**i**, **j**) show the median (central line), the 25–75% interquartile range (IQR) (box limits), the ±1.5 times IQR (Tukey whiskers), and all data points, among which the lowest and the highest points indicate minimal and maximal values, respectively.

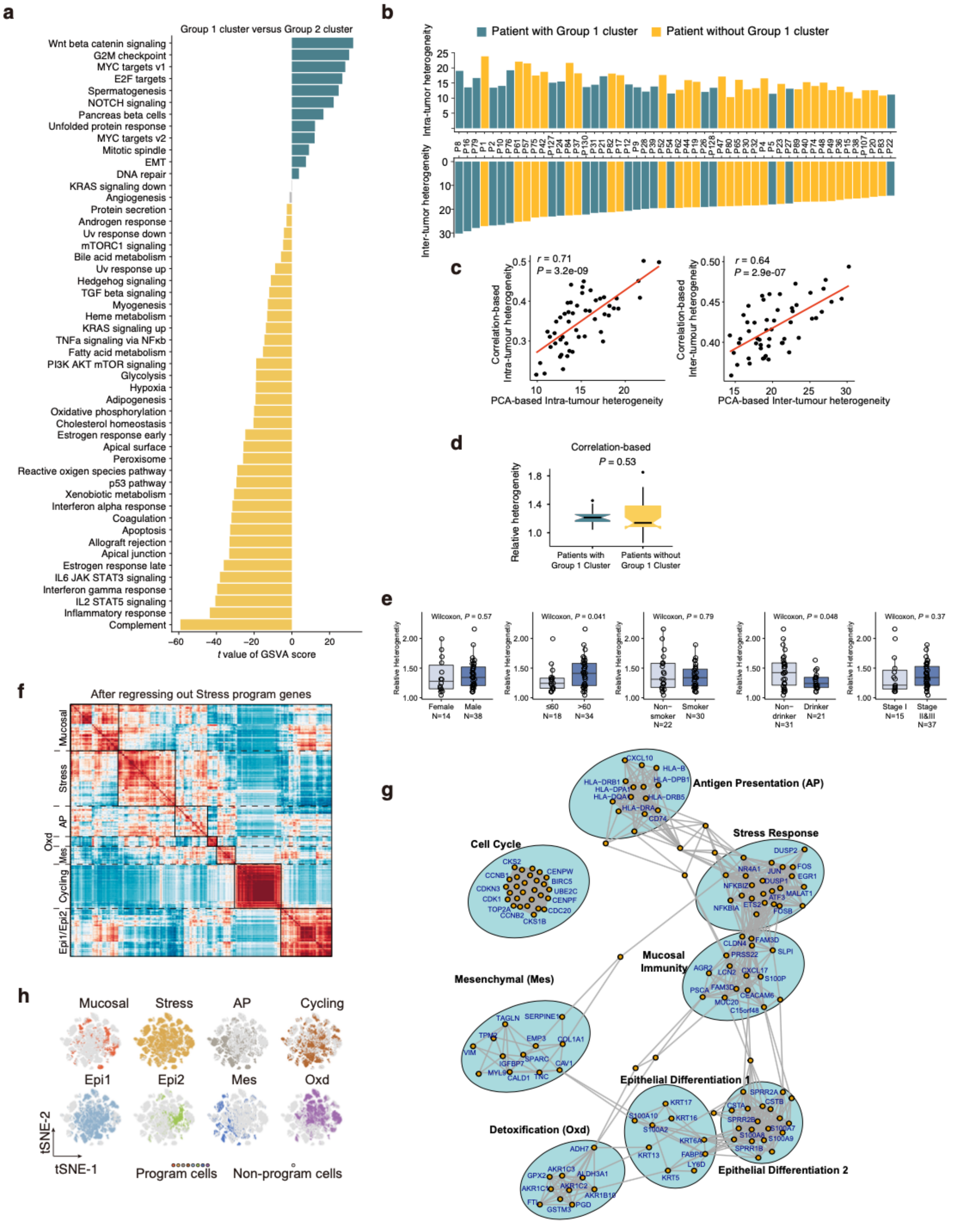

Supplementary Fig. 2 The heterogeneity and expression programs of epithelial cells. a Differences in pathway activities scored per cell by GSVA between Group 1 and Group 2 cluster. Shown are *t* values from the linear model. **b** Histogram showing the intra-tumor (*top*) and inter-tumor (*bottom*) heterogeneity levels of epithelial cells. Patients are ordered by the inter-tumor heterogeneity level. **c** Spearman correlation analysis for intra- and inter-tumor heterogeneity scores evaluated using PCA-based and Correlation-based methods for all samples. **d** Comparision of relative heterogeneity score for patients with (N = 21) or without (N = 31) Group 1 cluster using

Correlation-based method (two-sided Wilcoxon test). **e** Boxplots showing correlations between relative heterogeneity and clinical characteristics of patients. *P* values are derived from two-sided Wilcoxon tests. **f** Heatmap showing pairwise correlations of 381 modules derived from 52 tumors after regressing out Stress program genes. The common expression programs across tumors are aggregated into clusters. **g** Gene co-expression network analysis of the genes from 8 expression programs. **h** The distributions of each expression program. Cells expressing ≥ 70% of genes in a given program are defined as program cells otherwise are non-program cells. Box plots in (**d**, **e**) show the median (central line), the 25–75% interquartile range (IQR) (box limits), the ±1.5 times IQR (Tukey whiskers), and all data points, among which the lowest and the highest points indicate minimal and maximal values, respectively.

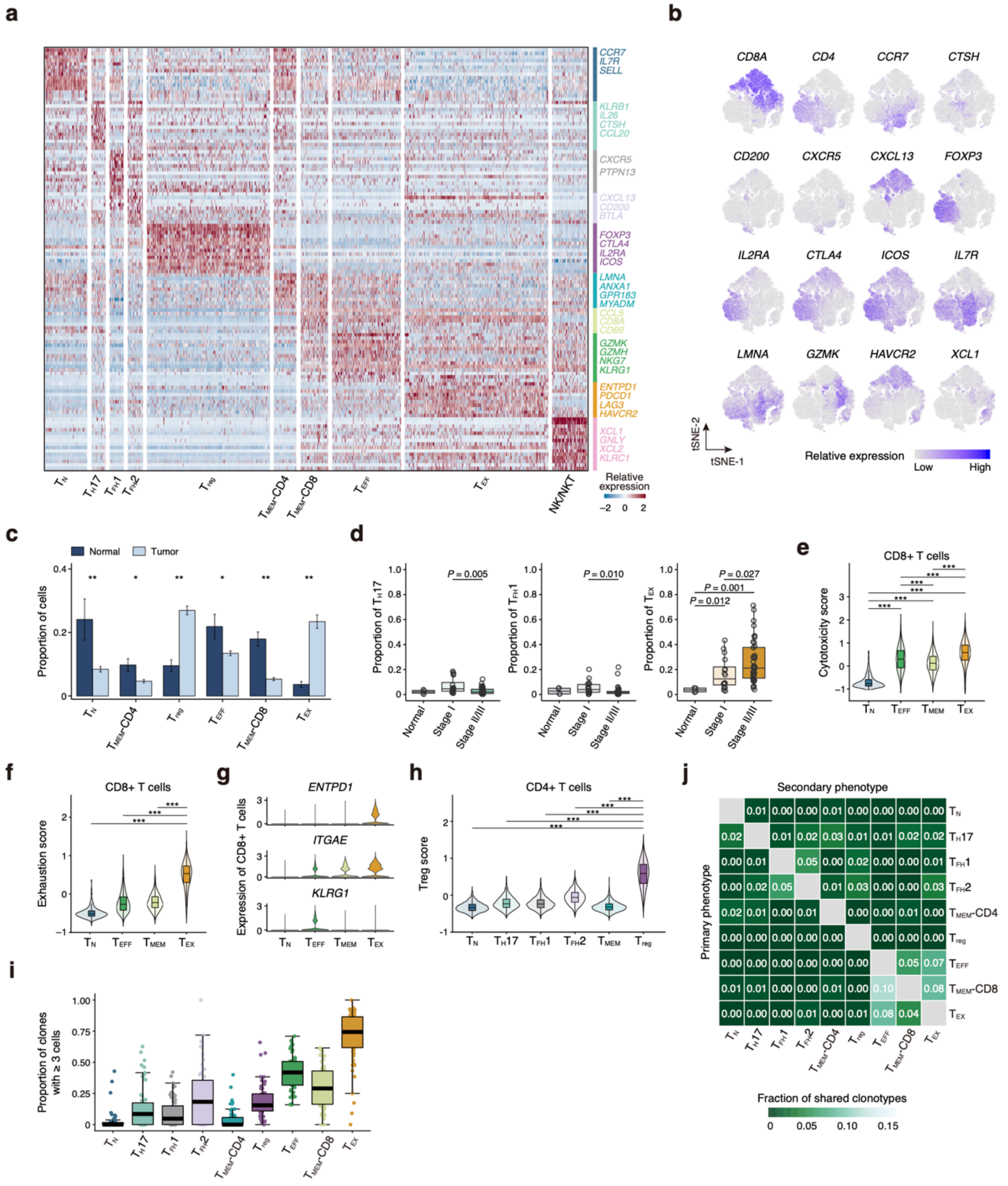

Supplementary Fig. 3 Characterization of T cell subtypes. a Heatmap showing the expression levels of the marker genes expressed by T cell subtypes. **b** tSNE plots of T cells with color-coded for expression levels of the classical markers. **c** Histograms showing the different distribution of subtype proportions between tumor (N = 60) and adjacent normal (N = 4) tissues. *P* values: 0.006 (T_N), 0.012 (T_{MEM}-CD4), 0.002 (T_{reg}), 0.034 (T_{EFF}), 0.001 (T_{MEM}-CD8), 0.002 (T_{EX}). Data presented as mean ± SEM (two-sided Wilcoxon tests). **d** Boxplots comparing proportion of cells in normal tissues (N = 4), stage I (N = 16) and stage II/III (N = 44) tumors (two-sided Wilcoxon tests). **e** The distribution of cytotoxicity scores in all CD8+ T cell subtypes. Cells from each subtype are N = 444 (T_N), 9,407 (T_{EFF}), 3,767 (T_{MEM}) and 19,633 (T_{EX}), respectively (two-sided Wilcoxon tests). **f** The distribution of exhaustion scores in all CD8+ T cell subtypes. Cells from each subtype are N = 444 (T_N), 9,407 (T_{EFF}), 3,767 (T_{MEM}) and 19,633 (T_{EX}), respectively (two-sided Wilcoxon tests). **g** Normalized expression levels of 3 marker genes of tumor-reactive T cells in CD8+ T cells. **h** The

distribution of Treg scores in all CD4+ T cell subtypes. Cells from each subtype are N = 1,818 (T_N), 1,941 (T_H17), 1,939 ($T_{FH}1$), 2,101 ($T_{FH}2$), 3,124 (T_{MEM}) and 16,801 (T_{reg}), respectively (two-sided Wilcoxon tests). **i** The boxplots showing proportions of cells from clones with ≥ 3 cells for each subtype using TCR analysis. Cells from each subtype are N = 4,911 (T_N), 1,232 (T_H17), 1,813 ($T_{FH}1$), 1,868 ($T_{FH}2$), 2,356 (T_{MEM}-CD4), 15,782 (T_{reg}), 7,265 (T_{EFF}), 3,215 (T_{MEM}-CD8), and 18,433 (T_{EX}), respectively. **j** Heatmap showing the fraction of clonotypes from a primary phenotype cluster (*row*) that are shared with other secondary phenotype clusters (*column*). Box plots in (**d**–**f**, **h**,**i**) show the median (central line), the 25–75% interquartile range (IQR) (box limits), the ±1.5 times IQR (Tukey whiskers), and all data points, among which the lowest and the highest points indicate minimal and maximal values, respectively. *P* values in (**c**, **e**, **f**, **h**) are derived from two-sided Wilcoxon tests; *: $P < 0.05$, **: $P < 0.01$, ***: $P < 0.001$. *P* values in (**e**, **f**, **h**) are all $< 2.2 \times 10^{-16}$.

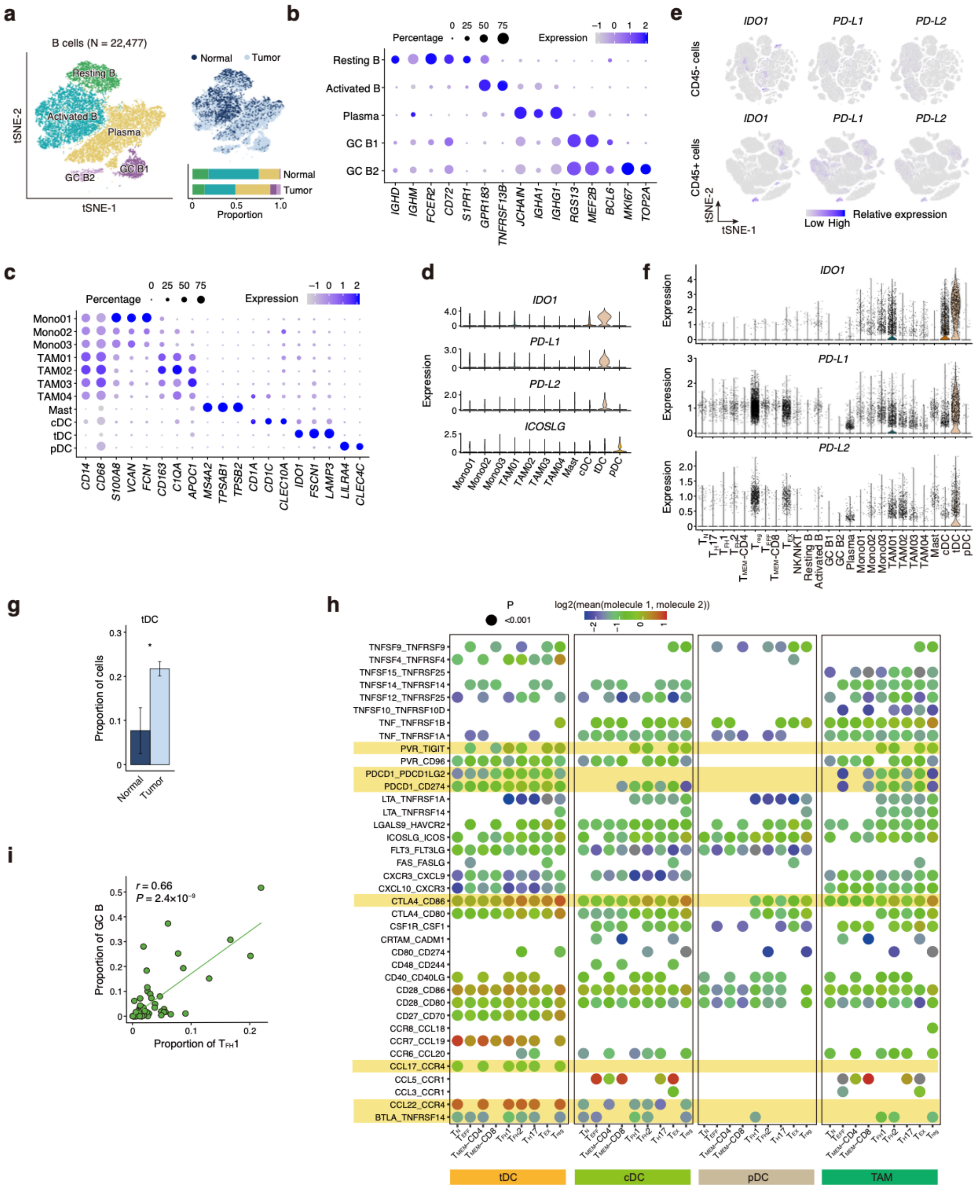

Supplementary Fig. 4 Characterization of B cell and myeloid cell subtypes. a tSNE plots of 22,477 B cells, colored by cell type (*left*) or tissue type (*top right*). The proportions of cell types in normal and tumor tissues are shown on bottom right (colors are the same as that in left panel). **b** Dotplot showing expression status of the marker genes for all B cell subtypes. Circle size indicates percentage of cells within each cell type expressing the marker; color intensity indicates mean marker expression level. **c** Dotplot showing expression status of the marker genes for myeloid cell subtypes. Circle size indicates percentage of cells within each cell type expressing the marker; color intensity indicates mean marker expression level. **d** Violin plots of the normalized expression levels of 4 immunosuppressive genes in all myeloid cell subtypes. **e** tSNE plots of CD45- and CD45+ cells with color-coded for the expression of *IDO1*, *PD-L1* and *PD-L2.* **f** Violin plots of the normalized expression levels of *IDO1*, *PD-L1* and *PD-L2* in all CD45+ cell subtypes. **g** Histograms showing

different proportion of tDC between tumor (N = 60) and adjacent normal (N = 4) tissues (two-sided Wilcoxon test; *: $P < 0.05$). P value: 0.026. Data presented as mean ± SEM. **h** The ligand-receptor interaction analysis between four immunoregulatory myeloid cell subtypes (tDC, cDC, pDC and TAM) and the T cell subtypes. **i** Spearman correlation between proportions of $T_{FH}1$ and GC B cells in TME.

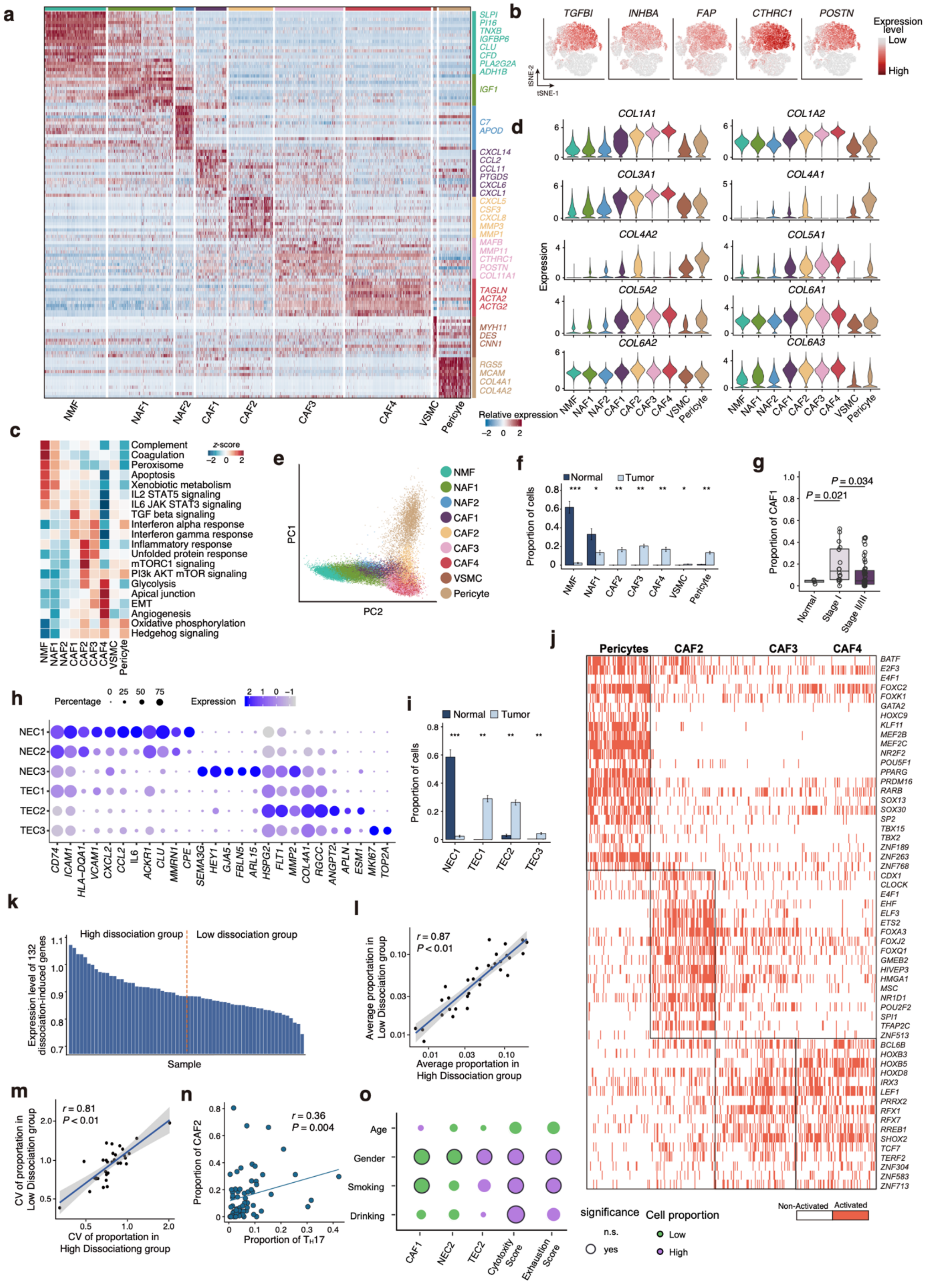

Supplementary Fig. 5 Characterization of fibroblasts, pericytes and endothelial cells. a Heatmap showing the expression levels of the marker genes in different fibroblast subtypes, pericytes and vascular smooth muscle cells (VSMC). **b** tSNE plots of fibroblasts with color-coded for the

expression of the classical CAF markers. **c** Heatmap showing differences in pathway activities scored by GSVA between different fibroblast subtypes, pericytes and VSMC. **d** Violin plots of the normalized expression levels of the collagen genes in different fibroblast subtypes, pericytes and VSMC. **e** The first two principal components of PCA on the transcriptome of fibroblasts, pericytes and VSMC colored by subtypes identified. **f** Histograms showing different proportions of fibroblast subtypes, pericytes and VSMC between tumor (N = 60) and adjacent normal (N = 4) tissues (two-sided Wilcoxon tests; *: $P < 0.05$, **: $P < 0.01$, ***: $P < 0.001$). *P* values: 0.001 (NMF), 0.022 (NAF1), 0.001 (CAF2), 0.001 (CAF3), 0.004 (CAF4), 0.027 (VSMC), 0.001 (pericyte). Data presented as mean ± SEM. **g** Boxplot comparing proportion of CAF1 in normal tissues (N = 4), stage I (N = 16) and stage II/III (N = 44) tumors (two-sided Wilcoxon tests). Box plots show the median (central line), the 25–75% interquartile range (IQR) (box limits), the ±1.5 times IQR (Tukey whiskers), and all data points, among which the lowest and the highest points indicate minimal and maximal values, respectively. **h** Dotplot showing expression status of the marker genes for all endothelial subtypes. Circle size indicates percentage of cells within each cell type expressing the marker; color intensity indicates mean marker expression level. **i** Histograms showing different proportions of endothelial subtypes between tumor (N = 60) and adjacent normal (N = 4) tissues (two-sided Wilcoxon tests; *: $P < 0.05$, **: $P < 0.01$, ***: $P < 0.001$). *P* values: 0.0004 (NEC1), 0.001 (TEC1), 0.002 (TEC2) , 0.002 (TEC3). Data presented as mean ± SEM. **j** SCENIC analysis of pericyte, CAF2, CAF3 and CAF4. Highly activated transcription factors (Fold change > 2, FDR < 0.05, two-sided Wilcoxon tests) are shown for each subtype. **k** Samples were divided into two groups, high or low dissociation group, aocroding to average expression levels of 132 dissociation-induced genes. **l** Spearman correlation between mean cell proportions in high dissociation group and low dissociation group. **m** Spearman correlation between coefficient of variation of cell proportions in high dissociation group and low dissociation group. **n** Spearman correlation between proportions of T_H17 cells and CAF2. **o** Bubble plot showing relationships between clinical factors and proportions of TME compositions as well as T cell scores. Compositions that are significantly increase or decrease in different clinical groups (two-sided Wilcoxon tests, $P < 0.05$) are indicated by purple or green, respectively. Circle size represents *P* value. Shaded regions in (**l**, **m**) indicate 95% confidence interval for the correlations.

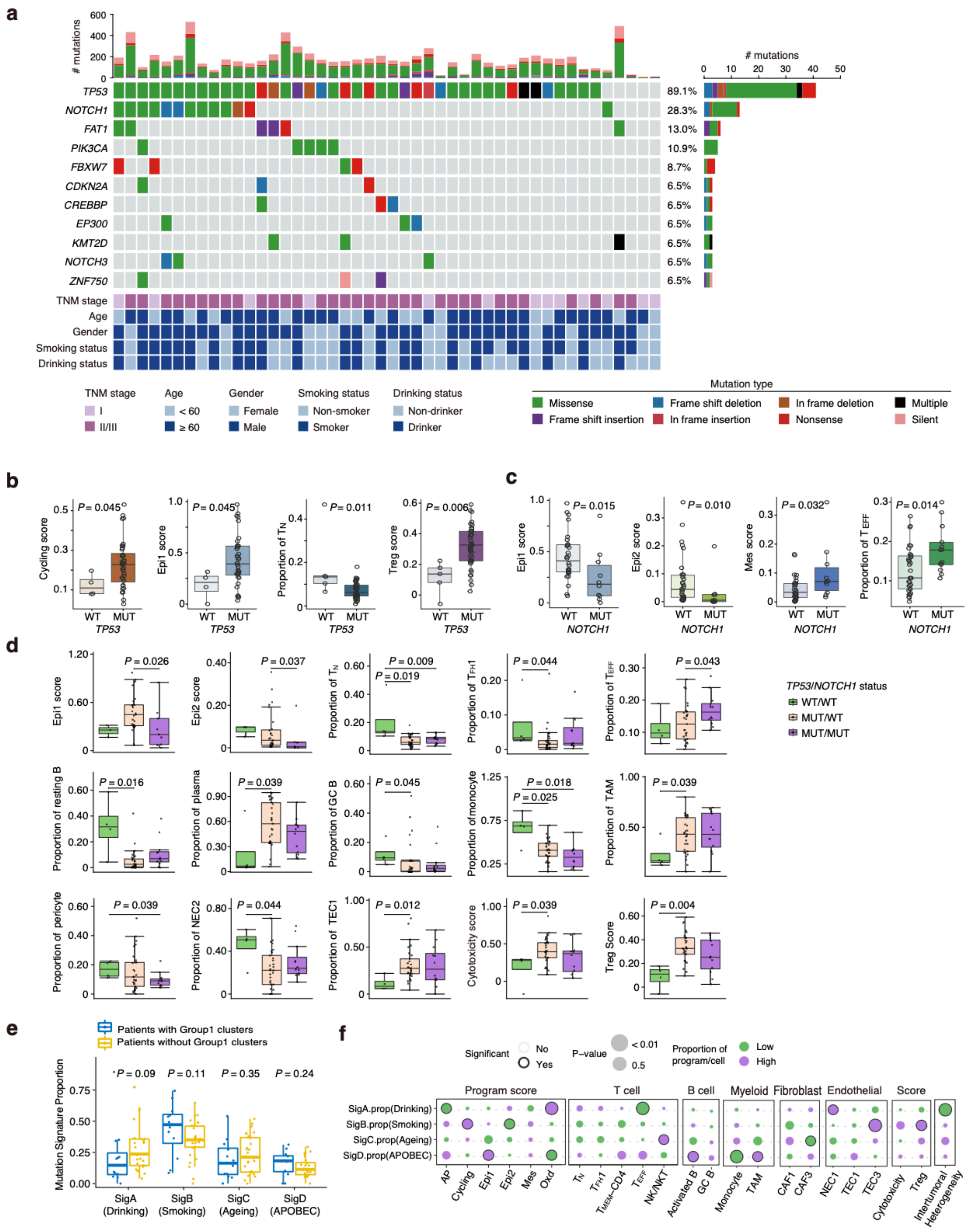

Supplementary Fig. 6 Landscape of somatic mutations in ESCC samples. a Putative driver genes are retained, each column denotes an individual tumor, and each row represents a gene. Samples are arranged to emphasize mutual exclusivity among mutations. Very top, total number of mutations (y axis) for each sample (x axis). Top, putative driver gene mutational landscape across analyzed ESCC samples with different mutation types color-coded differently. Bottom, key clinical parameters of each examined case. Right, percentage of mutation in ESCC samples while the horizontal axis represents total number of mutations for each gene. Clinical information and mutation types are shown by color as indicated. **b** Boxplots showing proportions of selected compositions, with comparisons between our patients with and without *TP53* mutation (N = 4

(WT) and 36 (MUT) for epithelial expression program score and N = 5 (WT) and 41 (MUT) for proportion and score of TME cells). *P* values are for two-sided Wilcoxon tests. **c** Boxplots showing proportions of selected compositions, with comparisons between our patients with and without *NOTCH1* mutation (N = 30 (WT) and 10 (MUT) for epithelial expression program score and N = 33 (WT) and 13 (MUT) for proportion and score of TME cells). *P* values are for two-sided Wilcoxon tests. **d** Boxplots comparing proportions of selected compositions in patients with different status of *TP53* and *NOTCH1* mutation (two-sided Wilcoxon tests). (N = 3 (WT/WT), 27 (MUT/WT) and 9 (MUT/MUT) for epithelial expression program score and N = 4 (WT/WT), 29 (MUT/WT) and 12 (MUT/MUT) for proportion and score of TME cells). **e** Boxplots comparing proportions of four mutational signatures between patient groups with (N = 16) or without (N = 24) Group1 Clusters. **f** Bubble plot showing relationships between four mutational signatures and program scores, proportions of TME compositions as well as T cell scores. Compositions that are significantly increase or decrease in mutated tumors (two-sided Wilcoxon tests, $P < 0.05$) are indicated by purple or green, respectively. Circle size represents *P*-value. Box plots in (**b**–**e**) show the median (central line), the 25–75% interquartile range (IQR) (box limits), the ±1.5 times IQR (Tukey whiskers), and all data points, among which the lowest and the highest points indicate minimal and maximal values, respectively.

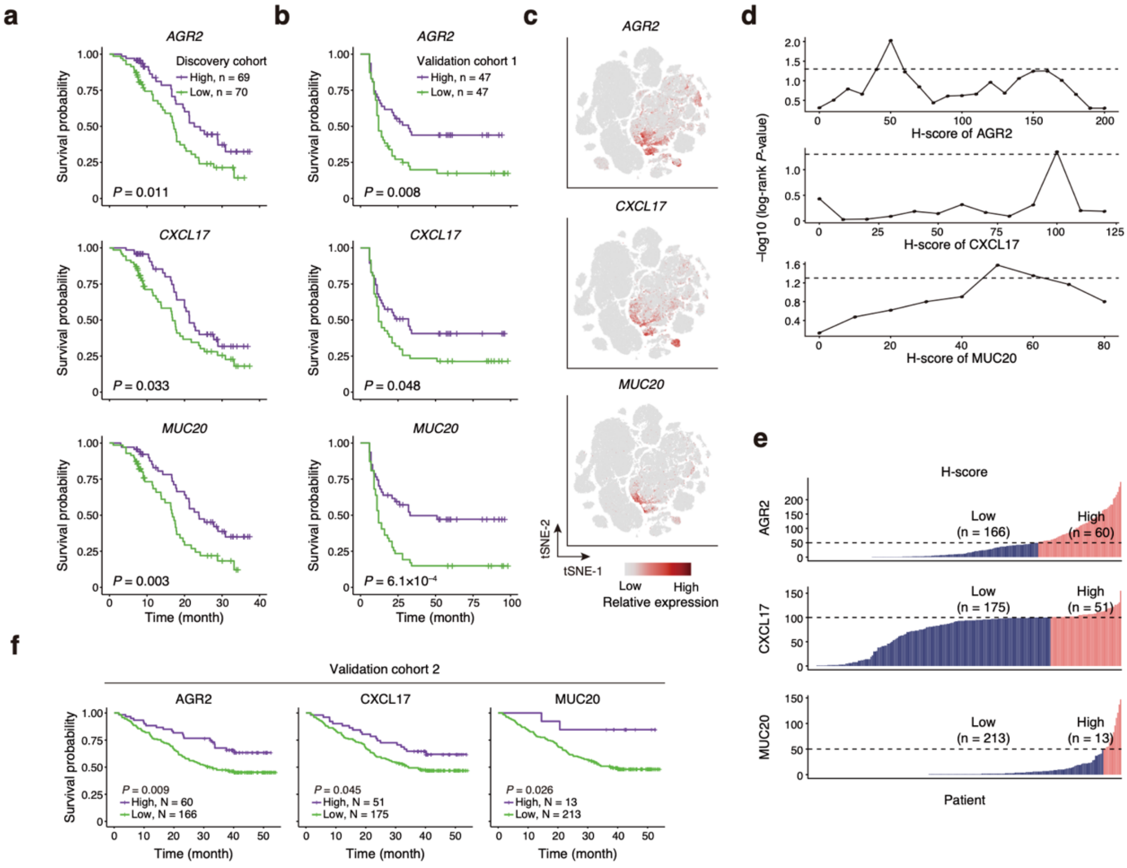

Supplementary Fig. 7 Analysis of correlations between expression levels of genes in epithelial cells and ESCC survival outcome. a and **b** Kaplan-Meier survival time analysis in Discovery cohort (**a**) and Validation cohort 1 (**b**) with patients grouped by expression levels of *AGR2*, *CXCL17* and *MUC20*. Purple lines: high expression groups; green lines: low expression groups. **c** tSNE plots showing the exclusive distributions of the 3 genes in epithelial cells among CD45- cells. **d** A minimum *P*-value analysis demonstrating potential H-score threshold values and corresponding *P*-values of AGR2, CXCL17 and MUC20. This graph presents the corresponding *P*-value of each two-sided log-rank test performed on a series of potential H-score thresholds of AGR2, CXCL17 and MUC20 to predict ESCC survival. The threshold with the smallest *P*-value is selected. The dashed lines indicate $P = 0.05$. **e** Histograms showing distributions of H-scores of AGR2, CXCL17 and MUC20 among ESCC samples. ESCC samples are grouped by the selected thresholds in (**d**). The dashed lines indicate the selected thresholds. **f** Kaplan-Meier survival curves of Validation cohort 2 grouped by H-score of AGR2, CXCL17 and MUC20, respectively. *P* values in (**a**, **b**, **f**) are derived from two-sided log-rank tests.
