# Supplementary material for: Dissecting esophageal squamous-cell carcinoma ecosystem by single-cell transcriptomic analysis
Source: Nat Commun. 2021 Sep 6;12:5291. doi: 10.1038/s41467-021-25539-x (PMC8421382; doi:10.1038/s41467-021-25539-x)
Supplement: Supplementary file 3 — Description of Additional Supplementary Files [file 41467_2021_25539_MOESM3_ESM.pdf]

Description of Additional Supplementary Files

File Name: Supplementary Data 1
Description: Clinical characteristics of patients in the scRNA-seq cohort, Discovery cohort, Validation cohort 1 and Validation cohort 2

File Name: Supplementary Data 2
Description: Quality measures of scRNA-seq experiments in CD45- and CD45+ cells

File Name: Supplementary Data 3
Description: Cell type counts

File Name: Supplementary Data 4
Description: Down-sampling analysis of changed cell types between adjacent normal tissues and ESCC tumors

File Name: Supplementary Data 5
Description: Eight epithelial expression program gene lists

File Name: Supplementary Data 6
Description: Cell subtype markers for T cells, B cells, myeloid cells, fibroblasts and endothelial cells derived from the analysis of scRNA-seq data

File Name: Supplementary Data 7
Description: T cell clones in ESCC tumors shared with adjacent normal tissues

File Name: Supplementary Data 8
Description: Associations between program gene expression levels and ESCC survival in Discovery cohort (N = 139) and Validation cohort 1 (N = 94)
