# Supplementary material for: Dissecting esophageal squamous-cell carcinoma ecosystem by single-cell transcriptomic analysis
Source: Nat Commun. 2021 Sep 6;12:5291. doi: 10.1038/s41467-021-25539-x (PMC8421382; doi:10.1038/s41467-021-25539-x)
Supplement: Supplementary file 12 — Reporting Summary [file 41467_2021_25539_MOESM12_ESM.pdf]

nature research

Corresponding author(s): Jianbin Wang & Chen Wu

Last updated by author(s): Jul 15, 2020

Reporting Summary

Nature Research wishes to improve the reproducibility of the work that we publish. This form provides structure for consistency and transparency in reporting. For further information on Nature Research policies, see our Editorial Policies and the Editorial Policy Checklist.

Statistics

For all statistical analyses, confirm that the following items are present in the figure legend, table legend, main text, or Methods section.

n/a	Confirmed	
☐	☒	The exact sample size (*n*) for each experimental group/condition, given as a discrete number and unit of measurement
☐	☒	A statement on whether measurements were taken from distinct samples or whether the same sample was measured repeatedly
☐	☒	The statistical test(s) used AND whether they are one- or two-sided<br>*Only common tests should be described solely by name; describe more complex techniques in the Methods section.*
☒	☐	A description of all covariates tested
☒	☐	A description of any assumptions or corrections, such as tests of normality and adjustment for multiple comparisons
☐	☒	A full description of the statistical parameters including central tendency (e.g. means) or other basic estimates (e.g. regression coefficient) AND variation (e.g. standard deviation) or associated estimates of uncertainty (e.g. confidence intervals)
☐	☒	For null hypothesis testing, the test statistic (e.g. *F*, *t*, *r*) with confidence intervals, effect sizes, degrees of freedom and *P* value noted<br>*Give P values as exact values whenever suitable.*
☒	☐	For Bayesian analysis, information on the choice of priors and Markov chain Monte Carlo settings
☐	☒	For hierarchical and complex designs, identification of the appropriate level for tests and full reporting of outcomes
☐	☒	Estimates of effect sizes (e.g. Cohen's *d*, Pearson's *r*), indicating how they were calculated

Our web collection on statistics for biologists contains articles on many of the points above.

Software and code

Policy information about availability of computer code

Data collection	Cell Ranger Single-Cell Software Suite (10x Genomics, version 2.1.0), cellranger vdj (10x Genomics, version 2.1.1)
Data analysis	R (version 3.5.1) with Seurat package (version 2.3.4), GSVA package (version 1.30.0), Limma package (version 3.38.3), Scanpy package (version 1.2.2) and estimate package (version 1.0.13)<br>CellphoneDB (version 2.0)<br>SCENIC python workflow (version 0.9.1)<br>BWA-MEM (version 0.7.17)<br>Picard (version 2.18.16)<br>GATK (version 4.0)<br>ANNOVAR (version 2017jun)<br>inForm (version 2.4.2)<br>BD FACSDiva (version 8.0.1)<br>Example scripts to process and analyze data is available at https://github.com/friedpine/scRNASeq_ESCC, detailed information will be available from the corresponding author upon reasonable request.

For manuscripts utilizing custom algorithms or software that are central to the research but not yet described in published literature, software must be made available to editors and reviewers. We strongly encourage code deposition in a community repository (e.g. GitHub). See the Nature Research guidelines for submitting code & software for further information.

Data

Policy information about availability of data

All manuscripts must include a data availability statement. This statement should provide the following information, where applicable:

- Accession codes, unique identifiers, or web links for publicly available datasets
- A list of figures that have associated raw data
- A description of any restrictions on data availability

The raw RNA and DNA sequencing data of this study have been deposited into the Gene Expression Omnibus (GEO) with accession number GSE160269 (https://www.ncbi.nlm.nih.gov/geo/query/acc.cgi?acc=GSE160269) and Sequence Read Archive with accession number SRP327447 (https://trace.ncbi.nlm.nih.gov/Traces/sra/?study=SRP327447), respectively. The raw sequencing data are also available from the Genome Sequence Archive of Beijing Institute of Genomics, Chinese Academy of Sciences with accession number HRA000195 (https://bigd.big.ac.cn/search/?dbId=&q=HRA000195).
Gene expression matrix of ESCC and paired adjacent normal samples are also available from the GEO with accession number GSE160269 (https://www.ncbi.nlm.nih.gov/geo/query/acc.cgi?acc=GSE160269). VCF files containing variants called from ESCC genomes have been deposited into the European Variation Archive with accession ID PRJEB41091 (https://www.ebi.ac.uk/ena/browser/view/PRJEB41091). Source data are provided with this paper. We obtained the hallmark gene sets from the MSigDB database (http://www.gsea-msigdb.org/gsea/msigdb/genesets.jsp?collection=H). The remaining data are available within the article, supplementary information or available from the corresponding author upon reasonable request.

Field-specific reporting

Please select the one below that is the best fit for your research. If you are not sure, read the appropriate sections before making your selection.

☒ Life sciences ☐ Behavioural & social sciences ☐ Ecological, evolutionary & environmental sciences

For a reference copy of the document with all sections, see nature.com/documents/nr-reporting-summary-flat.pdf

Life sciences study design

All studies must disclose on these points even when the disclosure is negative.

Sample size	Sample size was determined by the availability of recruited patients. Maximum number of available samples were used.
Data exclusions	This exclusion criteria were pre-established and generally used in 10x single cell assays. We removed genes that were detected in < 0.1% of all cells and filtered out cells with less than 500 detected genes or > 20% mitochondrial RNA content.
Replication	For immunohistochemistry, samples from 226 ESCC patients of the Validation cohort 2 were stained and similar staining results must be observed in over 3 visual fields. To ensure reproducibility of in vitro validation of tDCs' role in the ESCC TME (Figure 4i, j), two independent experiments were performed in each with three replicates. All attempts at replication were successful.
Randomization	Randomization is not applicable to the study design (single-cell and bulk RNA sequencing, whole-exome and whole-genome sequencing of tumor and non-tumor samples from diagnosed patients).
Blinding	Not applicable as there was no specific grouping.

Reporting for specific materials, systems and methods

We require information from authors about some types of materials, experimental systems and methods used in many studies. Here, indicate whether each material, system or method listed is relevant to your study. If you are not sure if a list item applies to your research, read the appropriate section before selecting a response.

Materials & experimental systems

n/a	Involved in the study	
☐	☒	Antibodies
☒	☐	Eukaryotic cell lines
☒	☐	Palaeontology and archaeology
☒	☐	Animals and other organisms
☐	☒	Human research participants
☒	☐	Clinical data
☒	☐	Dual use research of concern

Methods

n/a	Involved in the study	
☒	☐	ChIP-seq
☐	☒	Flow cytometry
☒	☐	MRI-based neuroimaging

Antibodies

Antibodies used	Antibody used for flow cytometry:<br>1; CD45-FITC (BD Biosciences; 555482; Mouse anti Human; monoclonal; dilution: 1:100; https://www.bdbiosciences.com/cn/

applications/research/stem-cell-research/cancer-research/human/fitc-mouse-anti-human-cd45-hi30/p/555482)
2; CCR7-PE (BioLegend; 353203; Mouse anti Human; monoclonal; dilution: 1:100; https://www.biolegend.com/en-us/products/pe-anti-human-cd197-ccr7-antibody-7498)
3; CD274-PerCP/Cy5.5 (BioLegend; 329737; Mouse anti Human; monoclonal; dilution: 1:100; https://www.biolegend.com/en-us/products/percp-cyanine5-5-anti-human-cd274-b7-h1-pd-l1-antibody-13403)
Antibody used for immunohistochemistry (IHC) staining:
1; AGR2 (Abcam; ab7
6473; Rabbit anti Human; monoclonal; dilution: 1:1000; https://www.abcam.cn/anterior-gradient-2-antibody-epr3278-ab76473.html)
2; CXCL17 (Proteintech; 18108-1-AP; Rabbit anti Human; polyclonal; dilution: 1:50; https://www.ptgcn.com/products/CXCL17-Antibody-18108-1-AP.htm)
3; MUC20 (Abgent, AP7830b; Rabbit anti Human; polyclonal; dilution: 1:100; https://www.abcepta.com/products/AP7830b-MUC20-Antibody-C-term)
Antibody used for inhibition of the PD1/PD-L1 pathway:
1; PD-L1 (Bio X Cell, BE0285; Mouse anti Human; monoclonal; dilution: 100 μg/ml; https://bxcell.com/product/invivomab-anti-human-pd-l1-b7-h1/)

Validation

All primary antibodies are tested and characterized as specific in human tissues, by the manufacturers, and are widely cited. Antibody-specific validations are available as indicated on the manufacturers' web page. Links are listed in the "Antibodies Used" section above. Antibodies were further validated in-house using relevant positive and negative controls

Human research participants

Policy information about studies involving human research participants

Population characteristics

The detailed clinical information of these patients was summarized in Supplementary Table 1. Patients received no chemotherapy or radiotherapy before endoscopy or surgery. Age and gender of the patients weren't restricted.

Recruitment

The cohort for scRNA-seq analysis comprised 60 patients from them fresh ESCC tumor, adjacent normal esophagus tissues (at least 5 cm away from tumor site) and peripheral blood samples were collected at the time of surgery in 2018 including 16 tumors at stage I, 18 tumors at stage II, and 26 tumors at stage III. For survival analysis, the Discovery cohort containing 139 ESCC patients were recruited between 2015 and 2017, Validation cohort 1 comprised 94 ESCC patients were recruited between 2010 and 2014 as described in our previous report and Validation cohort 2 consisting of 226 ESCC patients were obtained between 2015 and 2016. They were recruited after diagnosis at the Linzhou Cancer Hospital and Linzhou Esophageal Cancer Hospital (Henan Province, China) and were selected only based on ethnicity (Chinese) and sample availability. There were non potential self-selection bias or other selection biases.

Ethics oversight

This study was approved by the Institutional Review Boards of Cancer Hospital, Chinese Academy of Medical Sciences. Informed consent was obtained from each patient, and clinical information was collected from medical records.

Note that full information on the approval of the study protocol must also be provided in the manuscript.

Flow Cytometry

Plots

Confirm that:

- [x] The axis labels state the marker and fluorochrome used (e.g. CD4-FITC).
- [x] The axis scales are clearly visible. Include numbers along axes only for bottom left plot of group (a 'group' is an analysis of identical markers).
- [x] All plots are contour plots with outliers or pseudocolor plots.
- [x] A numerical value for number of cells or percentage (with statistics) is provided.

Methodology

Sample preparation

Cells from fresh ESCC tumors and their adjacent normal tissue samples were collected, details provided in method section.

Instrument

FACSAria flow cytometer (BD Biosciences)

Software

BD FACSDiva (v 8.0.1)

Cell population abundance

We determined the live cell counts using hemocytometer after sorting, and the cell concentration was adjusted to over 300 cells/μL before 10x assays.

Gating strategy

The FSC/SSC gating strategy was used to exclude cell debris and doublets. DAPI staining was used to exclude dead cells. CD45-FITC antibody staining was used as described in method section.

- [x] Tick this box to confirm that a figure exemplifying the gating strategy is provided in the Supplementary Information.
